# 

**Published:** 1914-05-30

**Authors:** 


### CONTENTS.

PAGE

**The Inspection of Public Institutions** ... ... 225

**A Year's Work at the Radium Institute** ... 226

**Hospital and Institutional News** ... ... ... 227

Our Hospital Sunday Appeal Number—Still Amongst the Rocks—Changes at North Evington Infirmary—Staff Resignations at King Edward VII.'s Hospital, Cardiff—Poor-Law Infirmaries and Insurance Certificates—A History of Bedlam—Head of the London Ambulance Service—The Dilke Memorial Scheme—Death of a Hospital Trustee—Guardians and the Feeble-minded — The Sex Instruction Controversy—The Value of the Festival Dinner—A Medical Superintendent's Mistake—Medical Staff Appointments in Glasgow—The Retrograde Policy of the Bath Guardians—Staines' New Cottage Hospital—Improvements at St. James's Infirmary — Relation of Environment to Worship—The Illness of Hospital Secretaries—The Pharmaceutical Council Election—Insured Women and the Drug Fund—American Institutions for Mental Defectives—The Welfare of the Blind : New Proposal—Radium for Glasgow—New Secretary of the Belgrave Hospital for Children—Staff Changes at Benenden Sanatorium—The Dispensers' Half-holiday—This Week's Drug Market.

**The Isolation Hospital Movement** ... ... 233

An Authoritative Institutional Textbook.

**More About Abderhalden's Reaction** ... ... 234

**The Duration of Pregnancy** ... ... ... 234

[See over.]

### CONTENTS—(*continued*).

| | PAGE |
|---|---|
| **Perfunctory Exercises in Schools** ... ... | 235 |
| The Inner Problem of Organised Games. | |
| **Cost of Hospital Treatment Claimed from a Doctor** ... ... ... ... ... ... ... | 236 |
| **Reports on Hospitals of the United Kingdom**—By SIR HENRY BURDETT, K.C.B., K.C.V.O. | 237 |
| North Evington Poor-Law Infirmary, Leicester. | |
| **London County Council Tuberculosis Scheme** | 239 |
| The Position of the Hospitals. | |
| **The London Panel Committee** ... ... ... | 240 |
| **The Editor's Letter-Box** ... ... ... ... | 241 |
| A Poor-Law Medical Officer and the Use of Brandy—Panel Doctors and Professional Cowardice—The Risk of Premature Burial—An Overlooked Factor in the Appeal for the Blind. | |
| **Round the World** ... ... ... ... ... ... | 242 |
| Fellow-Workers and their Doings. | |
| **The Practical Equipment of a Hospital** ... | 243 |
| The Ward Locker Competition: The Successful Designs. | |
| **Economy in the Generation and Use of Steam** ... ... ... ... ... ... ... ... | 244 |
| I. Sources of Waste in the Older Boilers. | |
| **Medical Appointments** ... ... ... ... ... | 244 |
| **The Art of Appealing to Working Men** ... | 245 |
| A Speech Delivered on Behalf of Ancoats Hospital. | |
| **The Department of Modern Nursing** ... .. | 247 |
| St. George's Hospital. | |
| **A Doctor's First Flight** ... ... ... ... | 249 |
| Relaxation by Aeroplane. | |
| **The National Medical Union (Official)** ... ... | 250 |
| Final Meeting of Provisional Federating Council. The Profession of Medicine. | |
| **The Institutional Worker** .. (Suppl.) | 1 |
| A Cheap, Efficient, Automatic Electric-Lighting System. | |
| Wastefulness of the Standard Gas Grill. | |

